# Supplementary material for: Synthesis and Application of Ion-Exchange Magnetic Microspheres for Deep Removal of Trace Acetic Acid from DMAC Waste Liquid
Source: Nanomaterials (Basel). 2023 Jan 27;13(3):509. doi: 10.3390/nano13030509 (PMC9918990; doi:10.3390/nano13030509)
Supplement: Supplementary file 1 [file nanomaterials-13-00509-s001.zip › nanomaterials-2143222-supplementary.pdf]

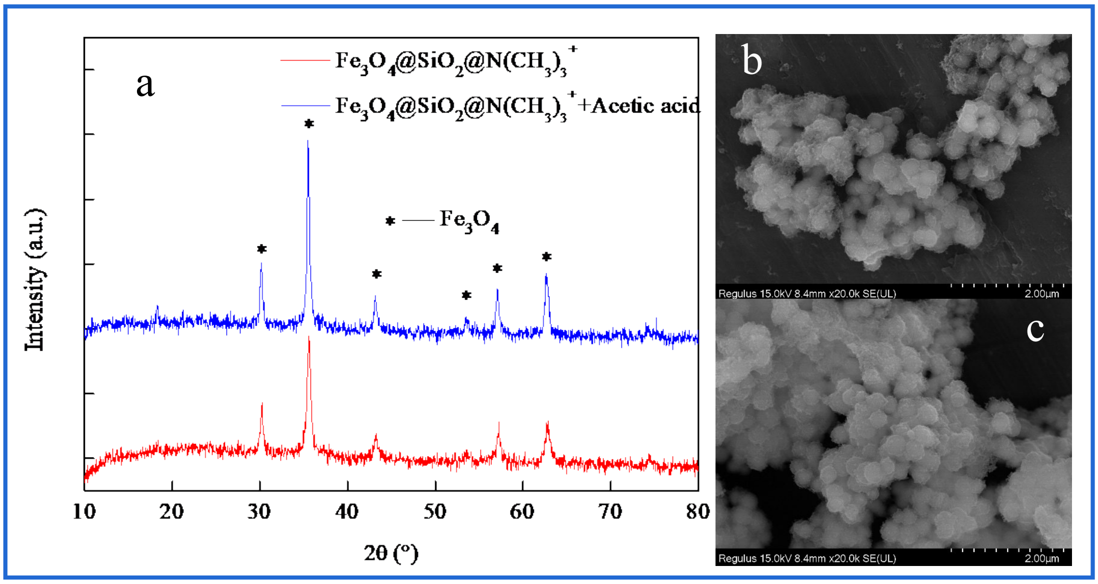

Figure S1. (**a**) FTIR spectra and SEM images of $Fe_3O_4@SiO_2@N(CH_3)_3^+$ (**b**) -before, (**c**)-after acetic acid adsorption.
